# Generation and Analysis of Ultrasound Images Using Plane Wave and Sparse Arrays Techniques

**DOI:** 10.3390/s18113660

**Published:** 2018-10-28

**Authors:** Nivaldo T. Schiefler, Joaquim M. Maia, Fabio K. Schneider, Acácio J. Zimbico, Amauri A. Assef, Eduardo T. Costa

**Affiliations:** 1 Department of Electronics, Federal Institute of Education, Science and Technology of Santa Catarina (IFSC), Joinville 89220-618, SC, Brazil; 2 Graduate Program in Electrical and Computer Engineering (CPGEI), Federal University of Technology—Paraná (UTFPR), Curitiba 80230-901, PR, Brazil; joaquim@utfpr.edu.br (J.M.M.); fabioks@utfpr.edu.br (F.K.S.); ajzimbico@gmail.com (A.J.Z.); amauriassef@utfpr.edu.br (A.A.A.); 3 Electronic Engineering Department and Graduate Program in Biomedical Engineering (PPGEB), Federal University of Technology—Paraná (UTFPR), Curitiba 80230-901, PR, Brazil; 4 Electrical Engineering Department and Graduate Program in Energy Systems (PPGSE), Federal University of Technology—Paraná (UTFPR), Curitiba 80230-901, PR, Brazil; 5 Biomedical Engineering Department of the School of Electrical and Computer Engineering (DEB/FEEC) and Biomedical Engineering Centre (CEB), State University of Campinas (UNICAMP), Campinas 13083-881, SP, Brazil; educosta@ceb.unicamp.br

**Keywords:** ultrasound, plane wave, sparse arrays, image reconstruction

## Abstract

Ultrasonic imaging is one of the most important techniques to help medical diagnosis. However, obtaining high quality images requires the acquisition, processing, and storage of a large amount of data. In this work, we evaluated a new ultrasound imaging technique based on plane wave and sparse arrays to increase the scan rate and reduce the amount of data amount to be stored. The performance of the proposed method was tested using simulated echo data (from Field II) and phantom data acquired using a Verasonics system equipped with a L11-4v linear array transducer. The tests were done using 128 elements for transmission and 128, 65, 44, and 23 elements sparsely distributed for reception. The simulated data were compared with images obtained with the Delay and Sum (DAS) method and the experimental data were compared with those acquired from Verasonics. The obtained results using the Full Width at Half Maximum (FWHM) criteria at −6 dB showed that the images generated by the proposed method were similar in terms of resolutions (axial and lateral) and contrast to the simulated and the Verasonics commercial ones, indicating that the sparse reception proposed method is suitable for ultrasound imaging.

## 1. Introduction

Currently, images of tissues and human organs might be acquired through several methods such as X-ray, tomography, magnetic resonance, and ultrasound [1]. Among the quoted methods, the ones that use the resonance and tomography techniques present an elevated quality on the final image exhibited by the equipment. However, the involved costs are higher, complicating the access to these kinds of exams for a major part of the population. Thus, since the beginning of the 2000s, the ultrasound (US) technique has been considered one of the most important imaging techniques to support clinical diagnosis in comparison with other methods that involve an invasive analysis with surgical procedures or images that are generated through ionizing radiations [2].

The US modality began to be used in the 50s for non-destructive testing focused on the evaluation of the physical properties of certain materials, which might reveal internal information and detect possible discontinuities or flaws [3,4,5]. In 1996, the US was already considered as the second most important modality for acquiring medical images to help in diagnosis, being surpassed only by the X-ray [3]. It is currently considered the main technique to evaluate soft tissues such as the liver, kidney, and others [6].

The final quality of the image obtained by US scanners is limited to the technical specifications of the equipment, the propagation of ultrasonic waves through the tissue to be analyzed [7], and the method used to reconstruct the images, which in the majority of the current equipment, is the traditional Delay and Sum (DAS) [8].

Many recent studies have focused on the reduction of the rate in which an ultrasonic transducer is excited or on the reduction of the number of signals to be received by the transducer elements (sparse arrays) to allow a higher frame rate (ultrafast image) to evaluate the moving structures in real time [9,10].

A new technique that has been used to increase the frame rate is the plane wave, where all elements are excited at the same time, taking a picture of the region of interest to be analyzed [11]. This technique reduces the number of scan lines needed, which consumes much memory to be stored and processing time. The acquisition of the signals in this case is done in a parallel way, and is limited to the number of channels receiving the signals and the acquisition rate of the hardware.

The US equipment should generate a good quality image. However, the reduction in the number of scan lines by the DAS technique does not improve the image quality due to the lack of energy resulting in a poor image quality [8]. To solve the image quality problem, new techniques must be used to process the signals in order to increase the frame rate and to allow better results in the lateral and axial resolutions [8].

Therefore, plane wave and sparse arrays have been used separately for many researchers to try to obtain better image quality with lower computational costs than the traditional beamforming techniques. However, to our knowledge, new proposals that combine both techniques have not yet been thoroughly investigated. So, this work proposes the use of a new technique to reconstruct US images based on plane waves to excite the transducer elements and the use of sparse arrays on the reception in order to increase the scan rate and to reduce the amount of data to be stored. The technique based on a coherent plane wave was included to increase the image quality due the use of sparse signals. The Stolt-migration technique was used as a processing base and an interpolation method was applied to reconstruct the ultrasound images. The proposed method was evaluated by the contrast analysis, lateral and axial resolutions for the images generated with data simulated on Field II [12,13], and the images generated by the US Verasonics system using the DAS method.

## 2. Materials and Methods

The traditional DAS technique uses several transmissions of US signals focused in one or more regions to scan the entire area to be analyzed and to form the scan lines that will be used to reconstruct the final image. This process is time-consuming and limits the frame rate to approximately 30 to 40 frames per second [10]. In order to avoid a large number of transmissions and reception of signals, the plane wave technique is being studied and used by some researchers [10,11,14,15,16].

### 2.1. Plane Wave

**Figure 1:**
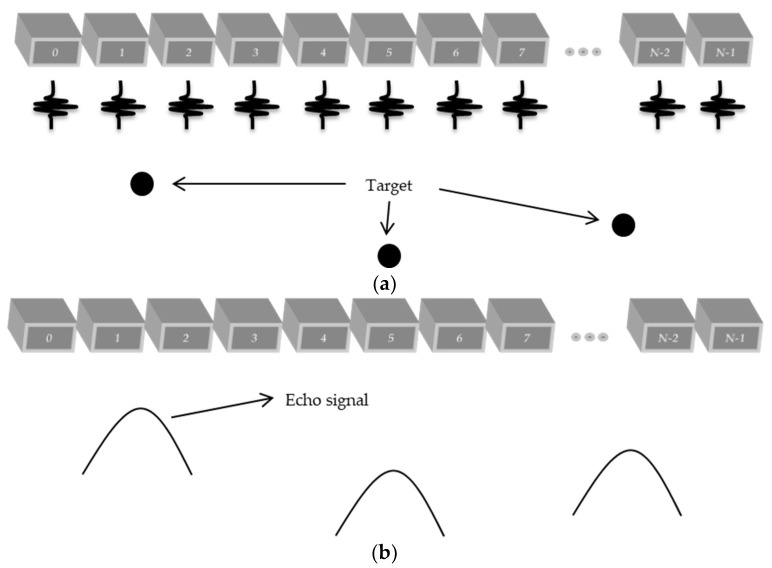
Plane wave technique: (**a**) All transducer elements are simultaneously excited to generate ultrasonic waves that reach the targets on the media to be analyzed; (**b**) Echo signals reflected on the targets and returning to all transducer elements.

Plane wave imaging differs from the conventional scanning systems by not using a focal point for the transmission of the US signal. Thus, the simultaneous excitation of all available elements in a certain transducer to transmit and collect ultrasonic signals is necessary. Figure 1a illustrates the principle of this technique in ultrasonic transmission and Figure 1b shows the return signal (echo signal) after reaching the targets.

**Figure 2:**
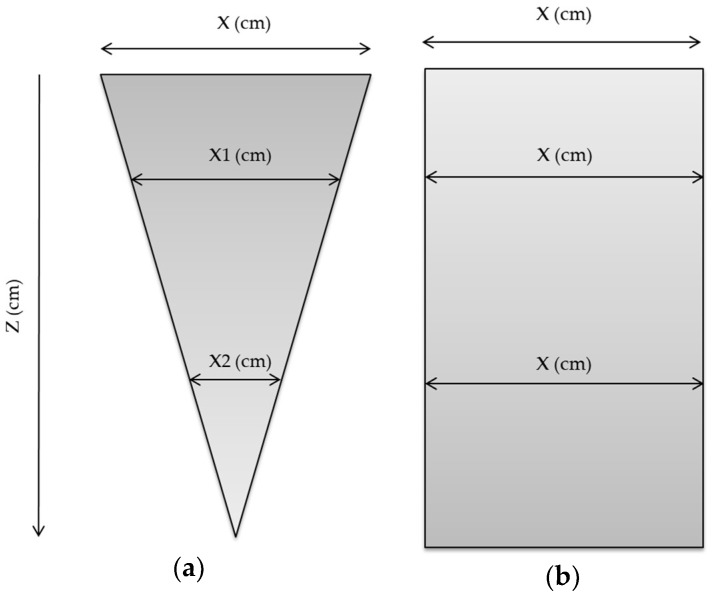
Coverage area for the (**a**) focused traditional DAS technique and (**b**) plane wave (without a focal point).

Due to the fact that all transducer elements can be simultaneously excited, the plane wave technique allows for the utilization of a single transmission circuit, reducing the hardware complexity, and becoming an advantage to the DAS method [15]. The targets echo signals return to the transducer and are received by all reception channels for a posteriori processing. This technique eliminates the need to form a scan line, increasing the scan area and generating a “photograph” of the target. Therefore, the system covers a wide area and not only a focal point, as can be seen in Figure 2. The disadvantage of this technique is the loss on the signal-to-noise ratio (SNR) and in the image resolution when compared to the DAS [15], making it necessary to have higher data processing and filtering to improve these parameters.

### 2.2. Coherent Plane Wave

**Figure 3:**
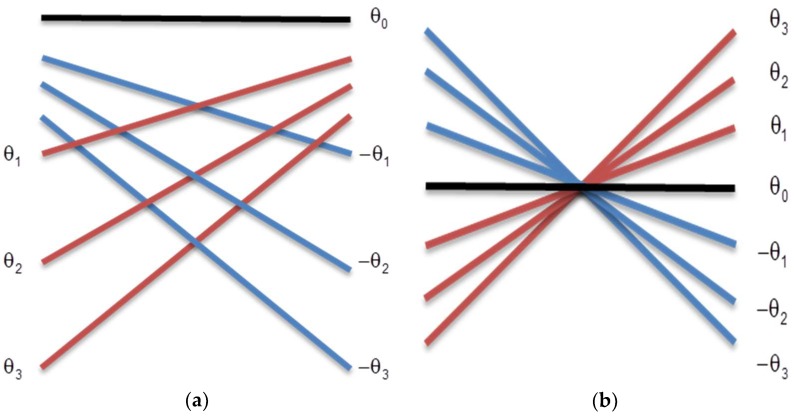
Coherent plane wave technique where all transducer elements are excited with different angles. (**a**) Separated excitation angles. (**b**) Representation of a focal point by the intersection of excitation angles.

A technique derived from plane waves used by Shen et al. [16] and that was proposed by Montaldo et al. [10], is the coherent plane wave. It uses the correlation of the acquired signals to generate a new database and forms the image after a new processing. Figure 3 shows this transmission process where, in the example, the ultrasonic pulse suffers an alteration due the inclusion of seven θ excitation angles.

**Figure 4:**
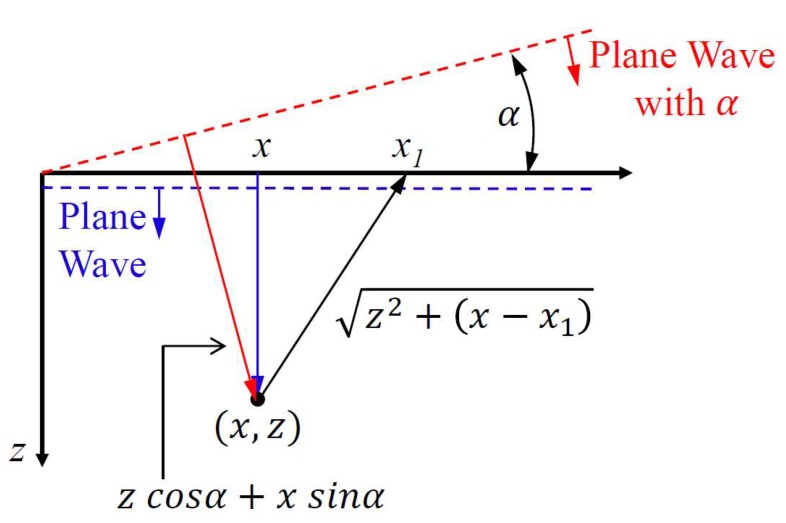
Projection of a target in plane wave without (blue dashed lines) and with angle α (red dashed lines) (Adapted from [10]).

A sequence of angular shots is applied (Figure 3a) allowing, in a certain way, the transmission focusing (Figure 3b). The image formation is done using several frames, with several excitation angles. Montaldo et al. [10] described the improvement on the lateral resolution, the contrast, and the SNR when compared with the results of the traditional B mode systems, and only the plane wave. The frame rate is limited by the number of angles required so that many shots demand a long processing time, therefore reducing its value. Figure 4 shows the typical configuration used to calculate the time delays for a target at the position (*x*, *z*) using plane wave [10]. The *x*-axis is the lateral direction and the *z*-axis is the axial direction in the imaging medium.

For a plane-wave excitation with angle α = 0 (blue dashed line in Figure 4), the traveling time τ between the excitation and the time of arrival of the ultrasonic echo to the transducer element placed in *x*_1_ is calculated by (1):

(1) $$\tau \left(x_{1},x,z\right)=\frac{z+\sqrt{z^{2}+{\left(x-x_{1}\right)}^{2}}}{c}\;$$

where *c* is the speed of sound in the medium and it is assumed to be constant (*c* = 1540 m/s). Therefore, all received *rf* (radiofrequency) data must be delayed by τ(*x*_1_, *x*, *z*) to the *x*_1_ direction before the coherent sum is calculated. By using the Equation (2), each point (*x*, *z*) of the image is obtained, where *a* represents the amount of elements of the transducer that contribute to the signal:

(2) $$s\left(x,z\right)=\int_{x-a}^{x+a}rf\left(x_{1},\tau \left(x_{1},x,z\right)\right)dx_{1}\;$$

Now, assuming that the red dashed line in Figure 4 represents the plane wave with angle α, the traveling time to the point (*x, z*) and return to the transducer element placed in *x*_1_ is given by Equation (3). Finally, the image is obtained as described in Equation (2), however, using the new values of τ [9,10]:

(3) $$\tau \left(\alpha ,x_{1},x,z\right)=\frac{z\cos\alpha +x\sin\alpha +\sqrt{z^{2}+{\left(x-x_{1}\right)}^{2}}}{c}\;$$

### 2.3. Sparse Arrays on the Reception

**Figure 5:**
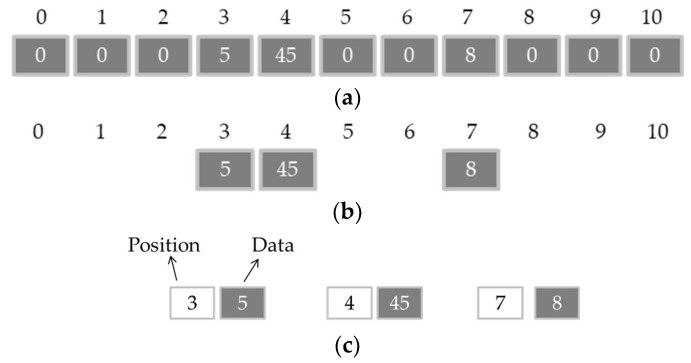

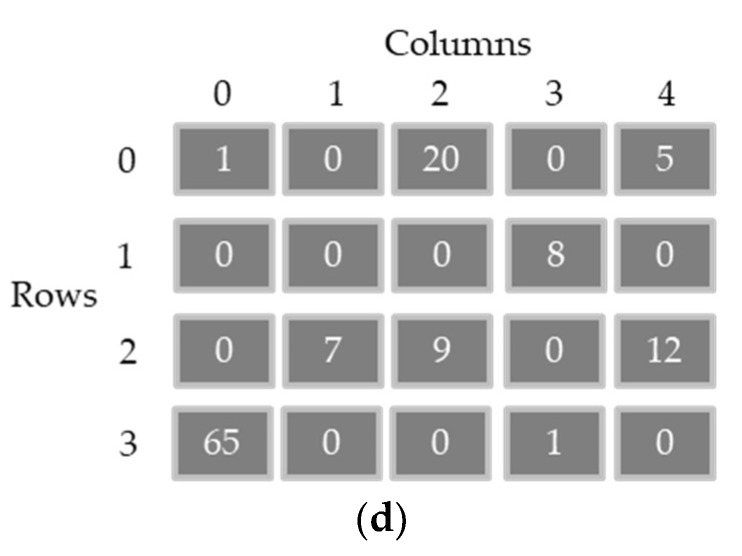
Examples of sparse arrays: (**a**) Sparse vector; (**b**) Relevant values of the sparse vector; (**c**) Column positions, followed by the valid value for them; (**d**) Sparse matrix.

During the reception and data processing for image reconstruction, it is possible to use, in some cases, only some of the total available elements of a vector or a matrix. A part of the existent elements might be ignored due the fact that it does not have a significant value, being represented by zeros on the matrix. When the amount of these values is relatively big and higher than the amount of the valid values, as exemplified in Figure 5, it is denominated as the sparse array [17,18]. Figure 5a shows a one-dimensional sparse array where there is a higher quantity of zeros than valid values. The storage of a relevant number of null values implies a waste of memory space in the data processing systems. Thus, the representation of relevant values could be done in a different way to allow a reduction of the vector or matrix size, and hence, a reduction in the storage space. This representation must consider only values different from zero. Figure 5b presents a sparse array considering only the relevant values and a possibility of a proper representation to save space in the memory, which would be to present a vector with the column positions, followed by the valid values for them, as shown in Figure 5c. The same can be done to a bi-dimensional array, represented as a sparse matrix (Figure 5d).

### 2.4. Stolt Migration

This technique is based on geologic studies to interpret seismic data and generate images of the region of interest [19]. This process has a function in the recovery of existing geometric relations on the reflection events caused by different acoustic impedances on the analyzed medium.

The original migration idea was to build a reflective map of the land through some seismic data recorded on a surface. The seismic signals are obtained by a sensor, which receives seismic waves superposed in all underground directions [20]. The migration term refers to the movement of the events observed on the spatial positions.

The migration is an inverse problem because the recorded waves are those transmitted and returned and they correspond to the localization of a certain reflector [20]. Based on Stolt’s proposal where a Fast Fourier Transform (FFT) is introduced, this method has been adapted for use in the plane wave mode [21,22]. The FFT used for US beamforming has been studied since the beginning of the 1980s [23]. The images can be transformed from the time domain to the frequency domain with the FFT, reducing the images’ coefficient sizes, therefore requiring a smaller memory space for the system [17,23] and allowing the generation of images in the plane wave mode to obtain the ultrafast acquisitions [24].

**Figure 6:**
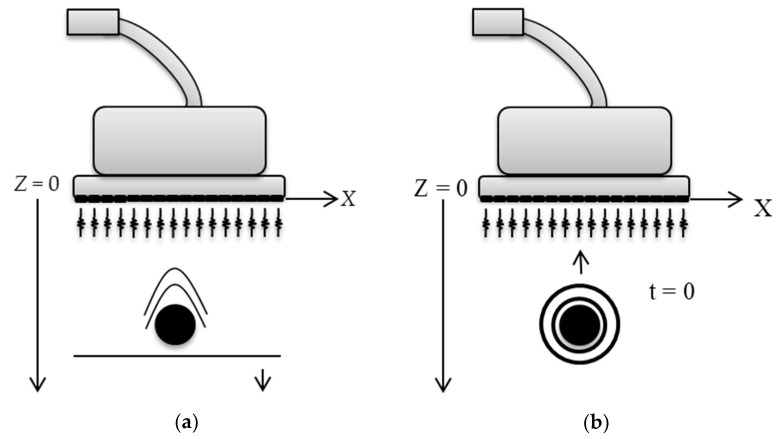
Representation of the echo signal using (**a**) the traditional model, and (**b**) according to the ERM model.

The method used in the work of Tarnec et al. and Garcia et al. was based on the Exploding Reflector Model (ERM), which assumes that all reflectors on the medium simultaneously explodes [21,22]. In this process, the wavefield considers that the explosion time is *t* = 0, becoming an acoustic issuing source. Figure 6a illustrates the traditional model used to represent the echo signal and Figure 6b, the ERM model.

Traditionally, each transducer element transmits the pulse signal and receives the echo in a pulse-echo operation mode. In this configuration, the traveling time (τ*_s_*) from the target at (*x_s_*, *z_s_*) to the transducer, depending on the position of each transducer element, can be calculated as in Equation (4) [21,22], where *c* is the speed of sound in the medium:

(4) $$\tau_{s}\left(x\right)=\frac{2}{c}\sqrt{{\left(x_{s}-x\right)}^{2}+z_{s}{}^{2}}\;$$

The Stolt migration is focused on the reverse wave propagation time, that is, the return time of the signal after the ERM explosion. Thus, it is enough to determine the signal’s return time to the sensor and not its total time (Equation (5)), where the *e* index means that the echo signal comes from the ERM position [21,22]:

(5) $$\tau_{e}\left(x\right)=\frac{1}{c_{e}}\sqrt{{\left(x_{e}-x\right)}^{2}+z_{e}{}^{2}}\;$$

In Equation (5), it is considered that the signal travels a single path to the transducer since the starting point is considered a signal generator source. Considering that (*x_s_*, *z_s_*) must be equal to (*x_e_*, *z_e_*), it is possible to state that when matching Equations (4) and (5), it will result in Equation (6), so only half of the propagation speed on the medium is necessary for the ERM method:

(6) $$c_{e}=\frac{c}{2}\;$$

The whole method described above and the sample routines are presented in [21,22]. So, the steps for image reconstruction using the Stolt migration technique are:

✓ Filtering of the raw signal
✓ Applying the FFT to the data
✓ Correction for the excitation angles
✓ Interpolation for a new scale
✓ Apply the inverse FFT to the data
✓ Additional filtering (optional)

In the interpolation step, all FFT data for a new scale is represented by Equation (7), proposed by Stolt, where *c_a_* represents the sound speed in the hyperbola peak (Equation (8)), and *k_x_* and *k_z_* are, the transducer position and frequency position spaced equally taking into account the power of 2 [21,22]. This equation considers the wave equation dispersion that has the relation between sampling frequency *f* and sound speed *c* in the medium [21,22]:

(7) $$f\left(k_{z}\right)=c_{a}.sign\left(k_{z}\right).\sqrt{k_{x}^{2}+k_{z}^{2}}\;$$

(8) $$c_{a}=\frac{\sqrt{2}}{2}c\;$$

In his work, Stolt has changed the variable $k_{z}$ to a new variable represented by Equation (9) that is determinate by equalizing Equations (4) and (5) with the first and second derivatives to find the maximum point:

(9) $$k_{z}=\sqrt{2}\frac{f}{c}\;$$

The interpolation must be done considering the original *f* scale that is determinate by sampling frequency and the new scale *f(k_z_)* calculated by Equation (7).

(10) $$rf_{ij}=rf_{i,j-1}+\frac{\left(rf_{i,j+1}-rf_{i,j-1}\right)\left(x_{1,j}-x_{1,j-1}\right)}{\left(x_{1,j+1}-x_{1,j-1}\right)}\;$$

**Figure 7:**
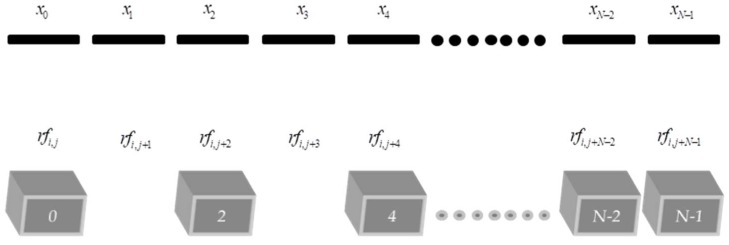
Representation of each transducer element (*x*_0_ *− x_N−_*_1_) and the sparse *rf* data to be interpolated (odd positions). The last right element is always valid.

The missing data calculation for the sparse cases were done using Equation (10) for a linear interpolation, considering the existing *rf* echo signal, the transducer size (*x*), the dimension of each element, and the spacing between the elements. Figure 7 shows the positioning of the transducer elements as well as the *rf* data, considering the sparse positions on the array. In this example, the odd positions were not considered, totaling 65 receiving elements for a 128-element transducer array (*N* = 128) including the last transducer element (*N* − 1) that was always valid in this work. This element was used as a reference for the interpolation algorithm. Sparse configurations with 44 and 23 receiving elements in the transducer were also tested in this work. In all sparse configurations tested, the last element would be zero when it is considering the numbers of sparse and actives elements, leading to an erroneous estimate of the last value. To solve this problem the last transducer element is considered always valid.

Table 1 summarizes all the processing steps applied in this study for generation of US images using plane wave and sparse arrays techniques. The circular averaging filter is optional and can be used to improve the resolution of *rf* data when small targets are present in the region of interest. These steps are performed for all frames and used for simulated and experimental data set.

### 2.5. Plane Wave Combined with the Sparse Array Method

**Figure 8:**
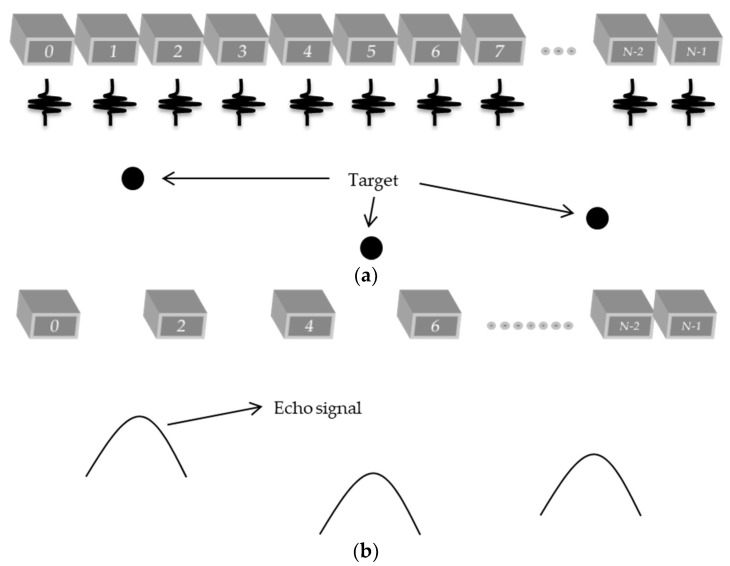
Plane wave with sparse arrays technique: (**a**) Transmission process of the ultrasonic pulse with all of the transducer elements; and (**b**) Reception of the echo signal using only the even elements including the last element (65 active elements).

On the plane wave with the sparse array technique proposed in this work, all of the transducer elements are excited, transmitting the ultrasonic pulses to the medium to be imaged (Figure 8a). On the reception, only some elements will receive the echoes’ signals. The example shown in Figure 8b considers that the even and the last right elements will receive the echoes returning from the targets in the medium. Therefore, the odd elements of the data matrix will be without information about the echo signal. Figure 8b represents the sparse condition with 65 active elements.

The fulfillment of the missing data was done through the linear interpolation technique, considering the adjacent elements of the sparse point for each sparse condition. The proposed method evaluation was done using the contrast analysis and lateral/axial resolutions for the isolated plane wave technique, plane wave with the transducer elements excited with different angles (ranging from −4.5° to +4.5°), and plane wave combined with sparse arrays (receiving data in 65, 44, and 23 transducer elements). All routines were developed and evaluated in Matlab R2010a (The Mathworks, Natick, MA, USA).

The simulations in this work were done using Field II and the real data were acquired using the Verasonics Vantage 128™ (Seattle, WA, USA) platform. In the simulation of the Field II data, it was defined as a phantom based on the available model in the application manual and was altered to suit the purpose of this work [12,25].

The phantom was defined with the following *xyz* dimensions: 40 × 10 × 90 mm. The sampling rate and the central frequencies of the transducer were 100 MHz and 6.25 MHz, respectively. The phantom was modeled with a solid point on the vertical position at 20 mm, a hyperechoic target (10 mm diameter), and an anechoic cyst (10 mm diameter) on the vertical positions located at 30 and 60 mm, respectively. For the plane wave simulation, a total of 50,000 points were used to generate the data for each setup.

For the experimental tests, a 6.25 MHz central frequency 128-element linear array transducer (model L11-4v) was used in the Verasonics system for data acquisition from an 84-317 Multipurpose Tissue/Cyst Ultrasound Phantom (Fluke Biomedical, New York, NY, USA). The sampling rate was set to 25 MHz. The simulation and the experimental specifications for the linear array transducer are presented in Table 2.

## 3. Results

The simulations in the Field II using the phantom described previously were done using plane waves with angle variation ranging from −1.5° to +1.5°, −3.0° to +3.0°, and −15.0° to 15.0°, with steps of 0.5°, 1.0°, and 5.0°, respectively, totaling seven angles for each range with five acquisitions for each angle. Other simulations with angle steps of 2.0°, 3.0°, and 4.0° were also undertaken and evaluated using the lateral and axial resolutions. In order to compare the results, the same phantom used to simulate the DAS technique was used for all tests.

For the experimental data through the Verasonics platform, two analyses were conducted in two distinct areas of the commercial phantom. The sparse condition used in the simulation and experimental data was defined with 65, 44, and 23 active elements. In the first experiment, it used the same angle variation from the simulation approach. In the second experiment, it used a sequence of angles ranging from −0.250 to +0.250°, with steps of 0.125°, totaling five angles with 10 acquisitions for each angle, and was used as a target representing an encapsulated cyst. The figure size for each simulation and the experimental tests were optimized to represent only the region of interest and not the full depth.

The evaluation of the image contrast (*ctr*) was done using Equation (11), described by [26], and by using an internal region of the cyst and the background marked on each image:

(11) $$ctr=20\log_{10}\frac{\left|\mu_{in}-\mu_{out}\right|}{\sqrt{\left(\sigma_{in}^{2}+\sigma_{out}^{2}\right)/2}}\;$$

where:

µ*_in_*: is the average value of the *rf* signal amplitude of the region of interest

µ*_out_*: is the average value of the *rf* signal amplitude outside the region of interest

σ^2^*_in_*: is the variance of the *rf* signal on the area of interest

σ^2^*_out_*: is the variance of the *rf* signal outside the area of interest

The mean square error (MSE), peak signal to noise ratio (PSNR), signal to noise ratio (SNR) and structural similarity index measure (SSIM), were used in this work to evaluate the image quality of the data processed using all sparse arrays setups [27,28,29].

### 3.1. Simulation Results

**Figure 9:**
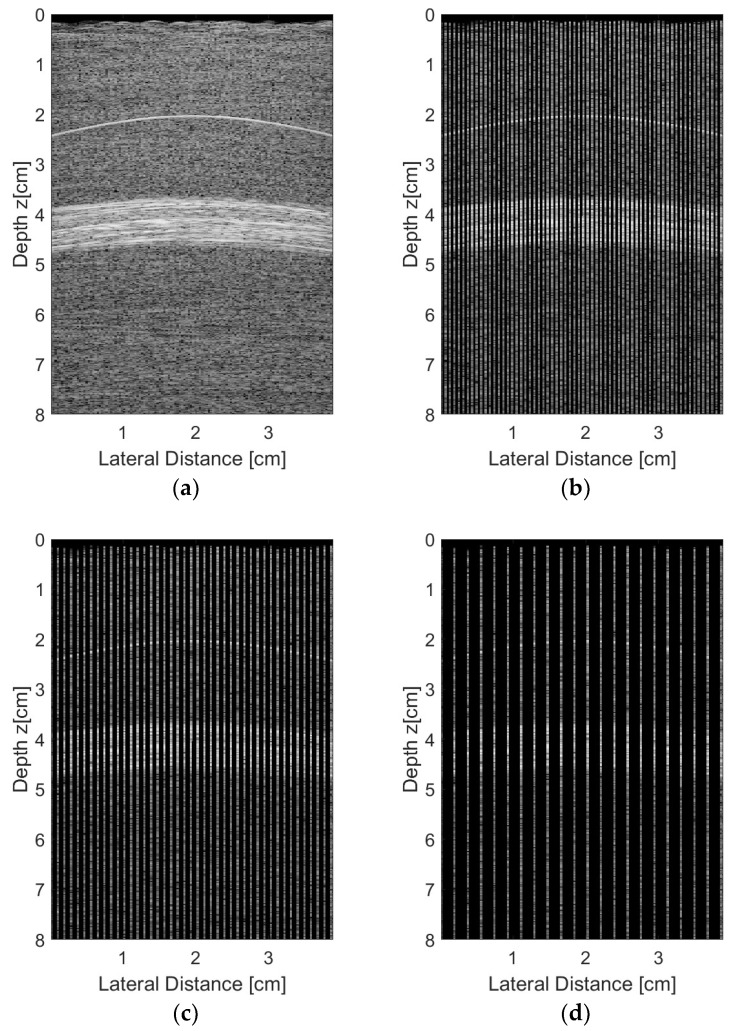
The *rf* raw signals for the plane wave excitation with an angle of 0° and (**a**) all 128 elements, (**b**) 65 elements, (**c**) 44 elements, and (**d**) 23 elements of the transducer receiving the data.

The simulated raw *rf* signals received by the transducer when excited with plane waves of zero degree angles (0°) are shown in Figure 9. The logarithmic compression was limited to −50 dB. Figure 9a shows the raw data with all 128 elements of the transducer receiving the signal and Figure 9b–d show the raw data for sparse conditions with 65, 44, and 23 active elements in the reception, respectively.

**Figure 10:**
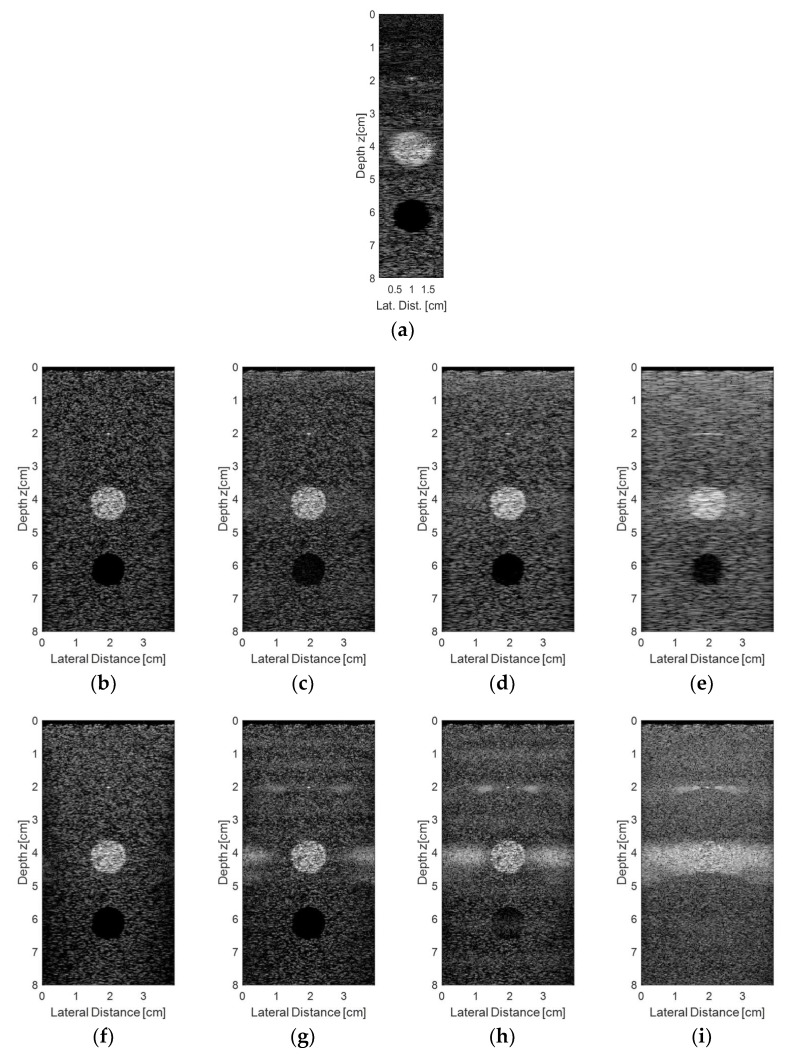
Simulated results for the raw *rf* signals acquired from the synthetic phantom by a linear 128 element transducer and processed with DAS and Stolt migration algorithms: (**a**) DAS, aperture of 64 elements, focal length at 2 cm; (**b**–**e**) Plane wave excitation with angles ranging from −1.5° to +1.5° (0.5° step) and the reception with (**b**) 128, (**c**) 65, (**d**) 44, and (**e**) 23 active elements; (**f**–**i**) Plane wave excitation with angles ranging from −15° to +15° (5.0° step) and reception with (**f**) 128, (**g**) 65, (**h**) 44, and (**i**) 23 active elements.

The simulated results for the raw *rf* signals acquired from the synthetic phantom described previously and processed with the Stolt migration and DAS algorithms are shown in Figure 10. Figure 10a shows the simulation results for the DAS method using the 128-element linear transducer, with an aperture of 64 elements and focal length at 2.0 cm. Figure 10b–e show the simulation results for the same transducer excited with plane waves with angles ranging from −1.5° to +1.5° (0.5° step) and the reception with 128 (Figure 10b), 65 (Figure 10c), 44 (Figure 10d), and 23 elements (Figure 10e), respectively. The simulation results for the plane wave with angles ranging from −15° to +15° (5.0° step) are shown in Figure 10f–i, with the same number of transducer elements used for data reception as in Figure 10b–e. It is possible to see that for small angles and sparse conditions (Figure 10c–e) there was an occurrence of some lateral spreading in the vicinities of the solid targets located at 2 cm and 4 cm depth. When the angle step was higher, it was possible to note that more scattering appeared in solid targets for sparse conditions (as shown in Figure 10g–i), decreasing the image quality and resolution.

**Figure 11:**
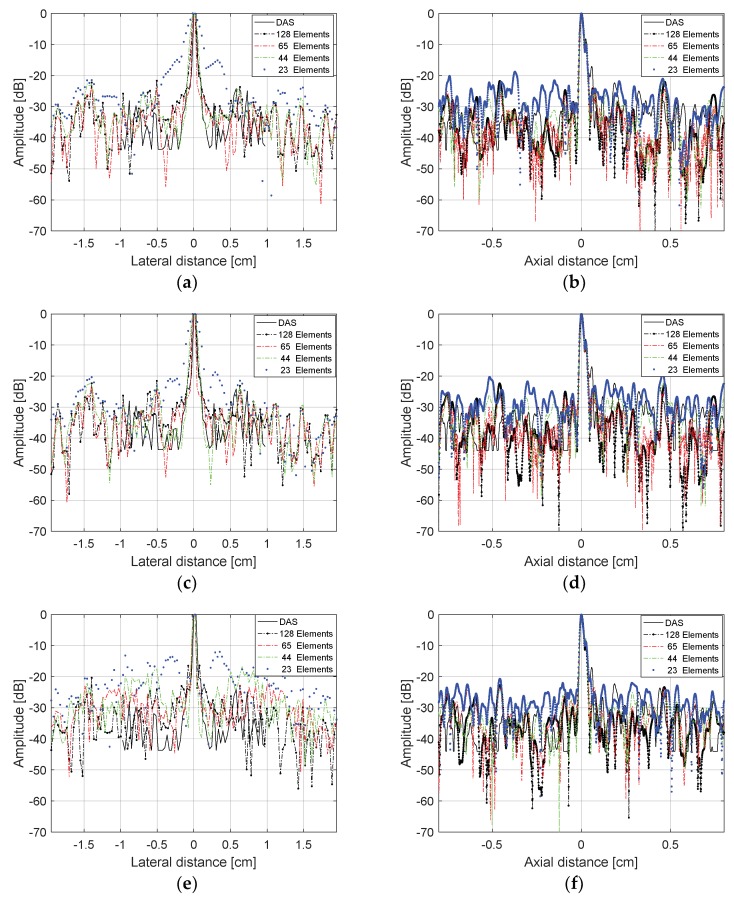
Lateral and axial resolution results for the target at 2.0 cm depth, shown in Figure 10, for the plane wave excitation and DAS method. Plane wave excitation with angles ranging (**a**,**b**) −1.5 to +1.5°, 0.5° step; (**c**,**d**) −3.0 to +3.0°, 1.0° step; (**e**,**f**) −15.0 to +15.0°, 5.0° step, and reception with 128 (non-sparse), 65, 44, and 23 active elements (sparse conditions).

The evaluation of the lateral and axial resolutions for the point located at 2 cm depth in Figure 10 were done using the Full Width at Half Maximum (FWHM) criteria at −6 dB [30]. Figure 11 shows the lateral and axial resolutions measured using the DAS method with the transducer excited with plane waves in three different angles ranges: −1.5° to +1.5° (0.5° steps), −3.0° to +3.0° (1.0° step), and −15.0° to +15° (5.0° step). In this case, we evaluated the reception with all 128 elements (non-sparse conditions) and with 65, 44, and 23 elements (sparse conditions). Analyzing Figure 11a,c,e, it is possible to see that there was a thinning of the central lobes and a soft reduction of the lateral lobes, decreasing the lateral resolution results when the sparsity was higher. Figure 11b,d,f show that the axial resolution results for the solid target at 2 cm depth, where it was possible to see that the central lobes had lower changes as the sparsity increased, keeping the axial resolutions almost constant.

Numerically, by the FWHM criteria, it is possible to compare the results acquired for the lateral and axial resolutions for the sparse configurations and with all elements from the transducer. Table 3 presents the lateral and axial resolutions, respectively, where it was possible to see a small difference between the lateral and axial resolution graphics. The traditional DAS method with 64 elements of aperture was used as a reference to compare with the Stolt migration method using the plane wave excitation at different angle ranges and receiving with all 128 elements (non-sparse mode) and with 65, 44, and 23 elements (sparse mode).

**Figure 12:**
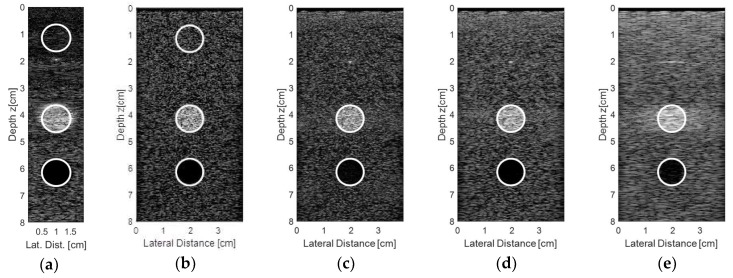
Contrast analysis for the solid and cyst targets and the background area at a 1 cm depth, as shown in (**a**,**b**), with the transducer excited using the DAS method (**a**) and with plane wave in an angle range of −1.5° to + 1.5°, step angles of 0.5°, and receiving the data with (**b**) 128, (**c**) 65, (**d**) 44, and (**e**) 23 elements.

The contrast and image quality analysis for Figure 12 using the DAS method and plane wave excitation with an angle range of −1.5° to +1.5°, 0.5° step, without any scattering close to the target at 2.0 cm, are shown in Table 3 and Table 4.

### 3.2. Experimental Results

**Figure 13:**
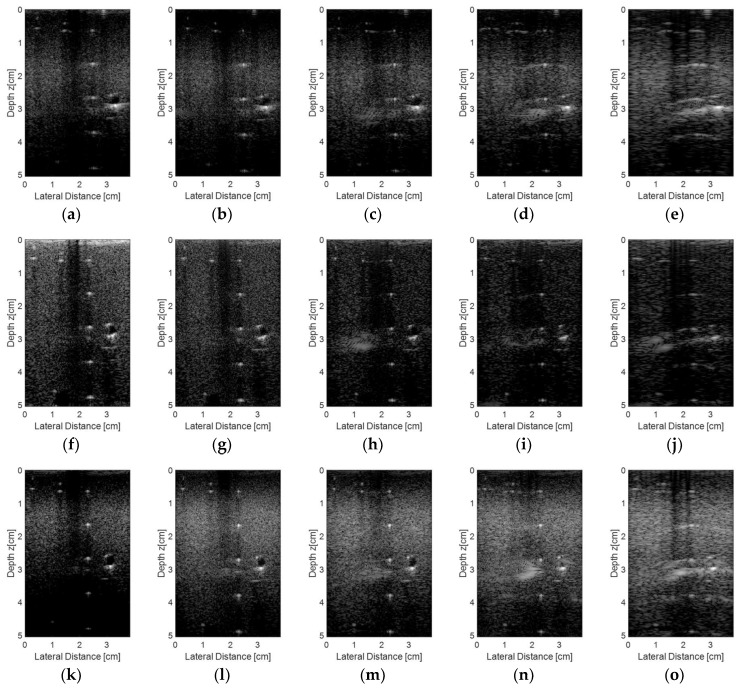
Images obtained from the commercial phantom using plane waves with seven angles and three angles steps: 0.5° (**a**–**e**), 1.0° (**f**–**j**), and 1.5° (**k**–**o**). The images were processed with the Verasonics VDAS method (**a**,**f**,**k**) and with the proposed method with all 128 elements (**b**,**g**,**l**), 65 elements (**c**,**h**,**m**), 44 elements (**d**,**i,n**) and 23 elements (**e**,**j**,**o**) receiving the data.

Figure 13 shows the experimental images obtained using the Verasonics Vantage 128™ system using the proprietary VDAS [31] method to process the data (Figure 13a,f,k) and with the linear L11-4v transducer excited with plane waves at different angles ranges (−1.5 to 1.5°, 0.5° step; −3.0° to +3.0°, 1.0° step; and −4.5 to 4.5°, 1.5° step). During the reception, we used all 128 transducer elements (Figure 13b,g,l), 65 elements (Figure 13c,h,m), 44 elements (Figure 13d,i,n), and 23 elements (Figure 13e,j,o).

**Figure 14:**
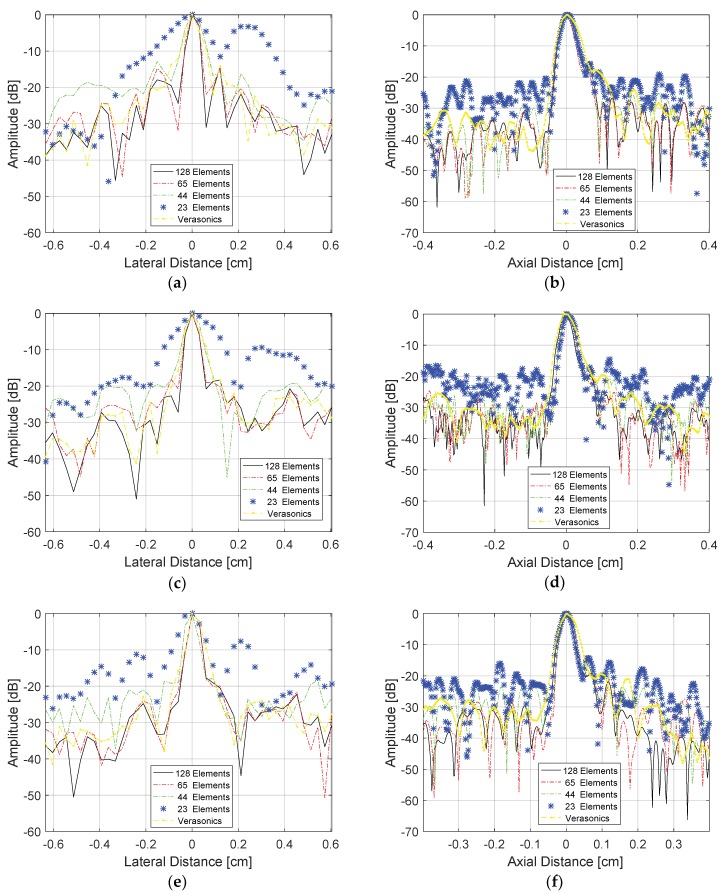
Analysis of the lateral resolutions (**left**) and axial resolutions (**right**) of the target at the depth of 1.7 cm (Figure 13) for angles (**a**,**b**) in the range −1.5 to +1.5° and 0.5° step; (**c**,**d**) −3.0 to +3.0°, 1.0° step; (**e**,**f**) −4.5 to + 4.5°, 1.5° step, respectively.

Figure 14 shows the lateral and axial resolution analysis for the target located at a depth of 1.7 cm as shown in Figure 13, and Table 5 shows the percentage errors. The transducer was excited with plane waves with angles in the range of −1.5 to +1.5° and 0.5° step (Figure 14a,b), −3.0 and +3.0° and 1.0° step (Figure 14c,d), and −4.5 to +4.5° with 1.5° step (Figure 14e,f). For all cases, it was considered that the data generated by the image from the Verasonics system (proprietary VDAS method) and the results with the data processed with all 128 elements of the transducer and the sparse array case with 65 active elements, which showed the best results with lower percentage errors for FWHM, as presented in Table 6. All images were processed for the dynamic range of −60 dB.

Analyzing the results shown in Table 6, it is possible to see that the lowest lateral error was obtained with angles in the range −3.0 to +3.0° and 1.0° step when compared with the reference image. However, the highest resolution was achieved with angles in the range −1.5 to +1.5° and 0.5° step, indicating that it is possible to obtain good quality images with a small angle variation.

Table 7 shows the image quality analysis in terms of Peak-SNR, higher SNR, lower MSE, and higher SSIM for the sparse condition with 65 active elements that showed the best results, using as a reference the images generated with all 128 elements being excited and receiving the data in the same angle range.

**Figure 15:**
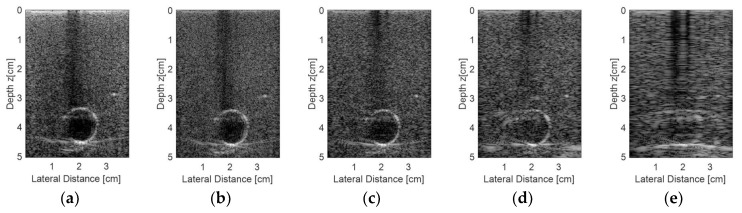
Images obtained from an encapsulated cyst of the commercial phantom with the transducer elements being excited with plane waves and angles in the range of −0.250° to +0.250°, 0.125° step. An average of 10 frames for each step were done for the processed images by (**a**) the Verasonics platform (using proprietary VDAS method), (**b**) using the Stolt migration method for all 128 elements and using the sparse array condition with (**c**) 65 active elements, (**d**) 44 active elements, and (**e**) 23 active elements.

Figure 15 shows the results for the second experiment, considering other regions from the commercial phantom and new acquisition data. A target was chosen with a diameter of 10 mm, representing an encapsulated cyst, and the transducer elements were excited with plane waves with angles in the range of −0.250° to 0.250° and 0.125° step, with 10 frames for each step. The resulting and processed images using the proprietary Verasonics VDAS method (Figure 15a) and using the Stolt migration method for all 128 elements being excited and receiving the data (Figure 15b) and for the sparse conditions with 65 (Figure 15c), 44 (Figure 15d), and 23 active elements (Figure 15e) are presented.

Analyzing the images shown in Figure 15, it was possible to see that the best result for the sparse condition was obtained with 65 active elements (Figure 15c) when compared with the Verasonics image (Figure 15a) and the image obtained with 128 active elements (Figure 15b).

**Figure 16:**
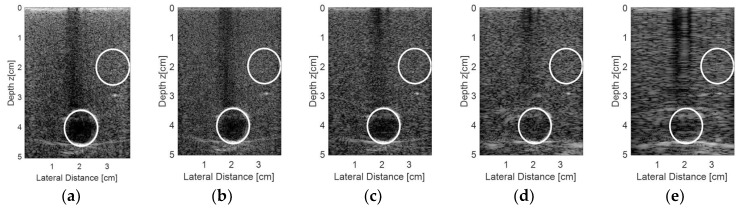
Regions of interest to quantify the contrast for the images obtained with (**a**) the Verasonics platform (DAS method), (**b**) processed with the Stolt migration method for all 128 elements and using the sparse array condition with (**c**) 65 active elements, (**d**) 44 active elements, and (**e**) 23 active elements.

Figure 16 shows the regions of interest for the contrast analysis of the images shown in Figure 15 and the results are presented in Table 8. The results of the sparse condition with 65 active elements were in very close agreement with the non-sparse condition (all 128 elements being excited and receiving the data).

Table 9 shows the image quality results in terms of higher similarity for the images shown in Figure 15 using the Verasonics image (Figure 15a) as a reference. The best results for the sparse condition was obtained with 65 active elements, with higher SNR and SSIM, and lower Peak-SNR and MSE.

## 4. Discussion

In this work, the generation of US images for the ultrafast modality with the combination of plane wave and sparse arrays techniques was evaluated. The utilization of sparse arrays allowed the reduction of data to be acquired and subsequently stored in temporary buffers for a posteriori processing.

Separately, the plane wave imaging and the sparse array imaging are well known and widely used techniques. However, in this work we combined and evaluated the two methods with different sparse conditions and several angle inclinations to improve the computational efficiency. The tests were done using 128 elements for transmission and 128, 65, 44, and 23 elements sparsely distributed for reception. The simulated data were compared with images obtained with the DAS method and the experimental data were compared with those acquired from Verasonics proprietary VDAS. The obtained results using the FWHM criteria at −6 dB showed that the images generated by the proposed method were similar in terms of resolutions (axial and lateral) and contrast, mainly using 65 active elements, to the simulated and the Verasonics commercial ones, indicating that the sparse reception proposed method is suitable for ultrasound imaging. Korukonda et al. [32] presented a work using the two methods for noninvasive vascular elastography. However, in that approach, only seven elements were applied in transmission and all 128 transducer elements were used to receive the *rf* data.

The simulations in Field II [12,25] showed good quality images with high lateral and axial resolutions. However, the simulated raw data had a higher number of samples (12,900 × 128) than the experimental data (2048 × 128) acquired with the Verasonics Vantage 128 platform, so that the same quality images were not possible to achieve with the experimental data.

Analyzing the results of the simulations in Field II as shown in Table 3, it was possible to see that the lowest percentage errors when compared to the DAS method for the lateral resolution (0.04%) and the axial resolution (−5.56%) were obtained with the transducers being excited with plane waves with angles from −15.0° to +15.0° (5.0° step) and −9.0° to +9.0° (3.0° step), respectively, and receiving the data in the sparse condition with 23 active elements. However, the images obtained (see Figure 10i, for example) showed a lot of scattering and it was not possible to visualize the targets adequately. Thus, although it presented a higher percentage error for the lateral and axial resolutions (5.51 % and −16.53 %, respectively), in the proposed method, images with the best quality were obtained with the transducer being excited with plane waves in an angle range of −1.5° to +1.5°, 0.5° step, and 65 active elements (Figure 10c) with lateral and axial resolutions of 0.4612 mm and 0.2348 mm, respectively.

The simulated results of the proposed method using the sparse condition with 65 active elements of a transducer with a total of 128 elements were comparable to the results obtained by other authors who used different techniques, targets, and phantoms to reach the best resolution.

Rindal and Austeng [33] obtained, for some sequences of plane waves and DAS methods, lateral resolutions of 0.56 to 0.82 mm and axial resolutions of 0.40 to 0.56 mm. With the usage of the minimum variance technique (MV), they obtained better results to the order of 0.07 to 0.12 mm for the lateral resolution and 0.41 mm to 0.42 mm for the axial resolution. They did not used the sparse condition in their work.

Using a DAS method, Mozaffarzadeh and collaborators [34] obtained lateral resolution values of 0.3931, 0.5825, and 0.8613 mm for a small depth of 25 mm. Even for a better resolution, according to the author, it was considered that the FWHM criteria for −3 dB, different from the metrics used in this work, which was −6 dB, as indicated by Harput et al. [30].

Comparing the results with other works that used the Stolt migration, Garcia et al. [22] showed that as the depth increased, the lateral resolution became worse. In comparative terms, for the same depth of the data simulated in this work (2 cm) they obtained lateral resolution values between 0.5 and 0.6 mm, considering all elements of the transducer, while in this work, the sparse condition was used.

The contrast analysis for the simulated data (Figure 12 and Table 4) in the sparse (65 active elements) and non-sparse (128 active elements) conditions were similar, being 7.03 dB and 6.49 dB for the solid target and 6.35 dB and 6.33 dB for the cyst, respectively. The same happened for the experimental results (Figure 16 and Table 8) with −1.66 dB for the sparse and −1.78 dB for the non-sparse condition. Analyzing the results shown in Table 4, it was possible to see that the obtained contrast was higher when using the DAS method to process the data from the solid target, and for the cyst, it was higher when using the plane wave method.

The image quality analysis for the plane wave method was done using the DAS method with 64 elements of aperture as a reference (non-sparse mode). Table 5 shows all of the analysis for the sparse condition processed with an angle range from −1.5° to +1.5° and 0.5° step. The sparse condition with 65 active elements showed the lowest Peak SNR and MSE and higher SNR, but the SSIM criteria was not so good. This happened due to the size of the images generated by each method (size for the DAS and size for the proposed method).

The contrast results in this work were close to the ones obtained by Matrone et al. [35], where the contrast was 1.3 and 1.8 dB. Analyzing the results obtained by Garcia et al. [22], it was possible to verify that the contrast values were close for variations up to 10 angles, regardless of the method used to reconstruct the images, and were comparable to those obtained in this work by using small angulation ranges. Furthermore, by evaluating the images generated by Verasonics and the ones processed by the sparse arrays method proposed here, it was possible to notice similar or even better results.

Experimentally, Kotowick et al. [36] obtained for a CIRS model 40 Multi-Purpose Multi-Tissue Ultrasound Phantom using DAS, plane wave compounding (PWC), and synthetic aperture (SA) in an adaptative system with lateral resolution values higher than 1.0 mm. In this work, we obtained a lateral resolution of 0.5815 mm (Table 6) with sparse arrays using 65 active elements and an angle range of −1.5° to +1.5°, 0.5° step. The axial resolution obtained in this work (0.4270 mm for the 65 active elements sparse condition) was also better than those obtained by the authors, which was 0.53 mm with SA and 0.64 mm for PWC.

Comparing the quality of the images generated by the proposed method in this work to the Verasonics VDAS method, it was possible to see that they were similar as it was possible to see all the phantom targets (Figure 13 and Figure 15). Increasing the angle range through the FWHM analysis, it was possible to verify that the resolution of the generated images was better in most cases, but some scattering began to appear, thus degrading the image quality.

The use of plane waves without angulation and sparse data in the reception was difficult in the reconstruction of detailed images, even with linear interpolation because there was a great loss of information. The effect was higher when the targets were in the direction of sparse elements. With the use of angulation, the effect of losing information was compensated and it was possible to reconstruct a detailed image, even when the sparse data were in the same direction of a small target. The tests showed good results when the angulation was increased, improving the attenuation between the central and secondary lobes. However, the scattering was a limiting factor for image quality when using sparse conditions with less than 65 active elements in the reception.

The Stolt migration method used in this work was shown to be proper, easy to implement, and fast to process the data. It was possible to clearly distinguish the points and cysts on the generated images. Even for the sparse case, the recomposition of the missing data using a linear interpolation presented good results.

## 5. Conclusions

Analyzing the obtained results in this work, it was possible to conclude that the proposed use of the sparse condition, with a reduction in the number of active elements of the transducer to receive the signal, will lead to a reduction in the hardware cost and complexity, mainly in the circuits aimed to amplify the signals, the high-speed analog to digital converters and filters.

The use of plane waves without angles to excite the transducer elements might result in a low-quality image, where it is difficult to identify the image contours, mainly when receiving data using sparse arrays. Thus, the acquisition of multiple frames and the angulation used to excite the transducer elements tends to compensate the information lost by the use of sparse conditions, improving the lateral and axial resolutions besides the contrast.

The use of new processing techniques for the reconstruction of US real time images has been established as a decisive factor to reach the ultrafast modality in USs. The migration method chosen to process the data depends on the inclusion of a direct or inverse FFT, which generates good results when combined with the acquisition of multiple frames and angulation.

The technique proposed in this work, using sparse arrays with a low number of frames and angles, was shown to be viable in generating ultrasound images with good quality and higher lateral and axial resolutions. Analyzing the image quality, our best results were obtained, for both simulation and experimental dataset, with 65 elements for the sparse condition and angle step of 0.5°. However, other new processing techniques to reduce the spreading should be included in the future to improve the final image quality.

## Figures and Tables

**Table 1:** Processing steps of the proposed method for generation of US images using plane wave and sparse arrays techniques.

Item	Description
I	The *rf* data are acquired in the sparse method, represented in a 2-D matrix form, by using appropriated apodization for each sparse condition
II	Linear interpolation is employed for the sparse condition selected as in Equation (10)
III	Band-Pass Filter with central *f_c_* = 6.25 MHz, inferior *f_i_* = 4 MHz and superior *f_s_* = 8 MHz cut-off frequencies is applied
IV	Compute *k_x_* and *k_z_* parameters
V	Compute *f* parameter in relation to the sampling frequency
VI	Compute the migrated *f(k_z_)* parameter as in Equation (7)
VII	Compute FFT 2D
VIII	Compute FFT Shift
IX	Angular correction is applied, as shown in Figure 4
X	Interpolate from *f* to *f(k_z_)*, the new scale
XI	Compute IFFT
XII	Apply a circular filter (optional)
XIII	Compute a log compression
XIV	Final Image

**Table 2:** Transducer parameters used for the simulation and experimental data acquiring (L11-4v).

Parameter	Simulating	L11-4v	Unity
Width	0.27	0.27	mm
Height	6.0	Not informed	mm
Kerf	0.035	0.030	mm
Central Frequency	6.25	6.25	MHz
Number of Elements	128	128	Elements
Number of Samples	12,900	2048	Per element

**Table 3:** FWHM—Lateral and axial resolution analysis and percentage errors for the images of the target located at 2.0 cm in the simulated phantom using the DAS method results as a reference to compare with the Stolt migration method.

Method	Angle Range (Degree)	Step (Degree)	Number of Elements Receiving	Lateral Resolution (mm)	Lateral Resolution Error (%)	Axial Resolution (mm)	Axial Resolution Error (%)
DAS	-	-	-	0.4881	-	0.2015	-
Stolt	−1.5 to +1.5	0.5	128	0.5779	−18.40	0.2165	−7.44
			65	0.4612	5.51	0.2348	−16.53
			44	0.9658	−97.87	0.2137	−6.05
			23	1.9772	−305.08	0.2162	−7.30
	−3.0 to +3.0	1.0	128	0.5707	−16.92	0.2155	−6.95
			65	0.4563	6.52	0.2344	−16.33
			44	0.9383	−92.24	0.2130	−5.71
			23	1.5811	−223.93	0.2130	−5.71
	−6.0 to +6.0	2.0	128	0.5408	−10.80	0.2180	−8.19
			65	0.5604	−14.81	0.2128	−5.61
			44	0.8249	−69.00	0.2138	−6.10
			23	1.0659	−118.38	0.2158	−7.10
	−9.0 to +9.0	3.0	128	0.5021	−2.87	0.2205	−9.43
			65	0.5206	−6.66	0.2152	−6.80
			44	0.6734	−37.96	0.2172	−7.79
			23	0.8202	−68.04	0.2127	−5.56
	−12.0 to +12.0	4.0	128	0.4604	5.68	0.2250	−11.66
			65	0.4868	0.27	0.2228	−10.57
			44	0.5633	−15.41	0.2175	−7.94
			23	0.6334	−29.77	0.2200	−9.18
	−15.0 to +15.0	5.0	128	0.4619	5.37	0.2279	−13.10
			65	0.4595	5.86	0.2251	−11.71
			44	0.4850	0.64	0.2169	−7.64
			23	0.4879	0.04	0.2282	−13.25

**Table 4:** Contrast analysis for the solid and cyst targets shown in Figure 12 with the data processed using the DAS method and plane wave without and with sparsity in the reception.

Method	Mode	Number of Elements (*e*) or Aperture (*a*)	Contrast for the Solid Target (dB)	Contrast for the Cyst (dB)
DAS	Non-sparse	64 (*a*)	9.59	5.34
Plane wave	Non-sparse	128 (*e*)	6.49	6.33
	Sparse	65 (*e*)	7.03	6.35
	Sparse	44 (*e*)	6.94	9.89
	Sparse	23 (*e*)	6.45	11.18

**Table 5:** Quality metrics analysis for images processed with angles range from −1.5° to + 1.5° and 0.5° step using the DAS method as reference.

Number of Elements	Peak-SNR (dB)	SNR (dB)	MSE (%)	SSIM
128	−19.81	12.31	95.76	0.04
65	−19.63	12.49	91.88	0.01
44	−20.31	11.82	107.31	0.03
23	−21.68	10.45	147.12	0.02

**Table 6:** FWHM—Lateral and axial resolution analysis and percentage error for the images of the target located at 1.7 cm in the phantom.

Number of Elements	Angle Range (Degree)	Step (Degree)	Lateral Resolution (mm)	Error (%)	Axial Resolution (mm)	Error (%)
Verasonics 128	−1.5 to +1.5	0.5	0.7218		0.5911	
128			0.5239	27.42	0.4312	27.05
65			0.5815	19.44	0.4270	27.76
44			0.8740	−21.09	0.4353	26.36
23			1.8974	−162.87	0.4301	27.24
Verasonics 128	−3.0 to +3.0	1.0	0.7188		0.5412	
128			0.6051	15.82	0.3738	30.93
65			0.6178	14.05	0.3736	30.97
44			0.7903	−9.95	0.3860	28.68
23			1.9524	−171.62	0.3894	28.05
Verasonics 128	−4.5 to + 4.5	1.5	0.6334		0.5522	
128			0.5491	13.31	0.3852	30.24
65			0.5390	14.90	0.4016	27.27
44			0.5969	5.76	0.3935	28.74
23			1.1101	−75.26	0.4013	27.33

**Table 7:** Image quality values of Peak-SNR, SNR, MSE, and SSIM obtained with 65 active elements being excited in a different angle range and using as a reference the images obtained with all 128 active elements and the same angle ranges.

Number of Elements	Angle Range (Degree)	Step (Degree)	Peak-SNR (dB)	SNR (dB)	MSE (%)	SSIM
65	−1.5 to +1.5	0.5	−8.18	26.74	6.58	0.77
65	−3.0 to +3.0	1.0	−11.93	22.67	15.61	0.60
65	−4.5 to +4.5	1.5	−11.35	22.92	13.64	0.66

**Table 8:** Contrast from circular areas for the images shown in Figure 16.

Number of Elements	Contrast (dB)
Verasonics 128	2.21
128	−1.78
65	−1.66
44	−4.84
23	−6.46

**Table 9:** Image quality values with the Verasonics image of the reference to cyst images.

Number of Elements	Angle Range (Degree)	Step (Degree)	Peak-SNR (dB)	SNR (dB)	MSE (%)	SSIM
128	−0.250 to +0.250	0.125	−21.83	11.86	152.49	0.27
65			−21.75	11.92	149.65	0.18
44			−21.86	11.77	153.44	0.11
23			−22.20	11.48	165.95	0.04
